# Supplementary figures and images for: Role of the type 6 secretion system on apoptosis and macrophage polarization during Burkholderia pseudomallei infection
Source: PLoS Negl Trop Dis. 2024 Oct 15;18(10):e0012585. doi: 10.1371/journal.pntd.0012585 (PMC11508161; doi:10.1371/journal.pntd.0012585)

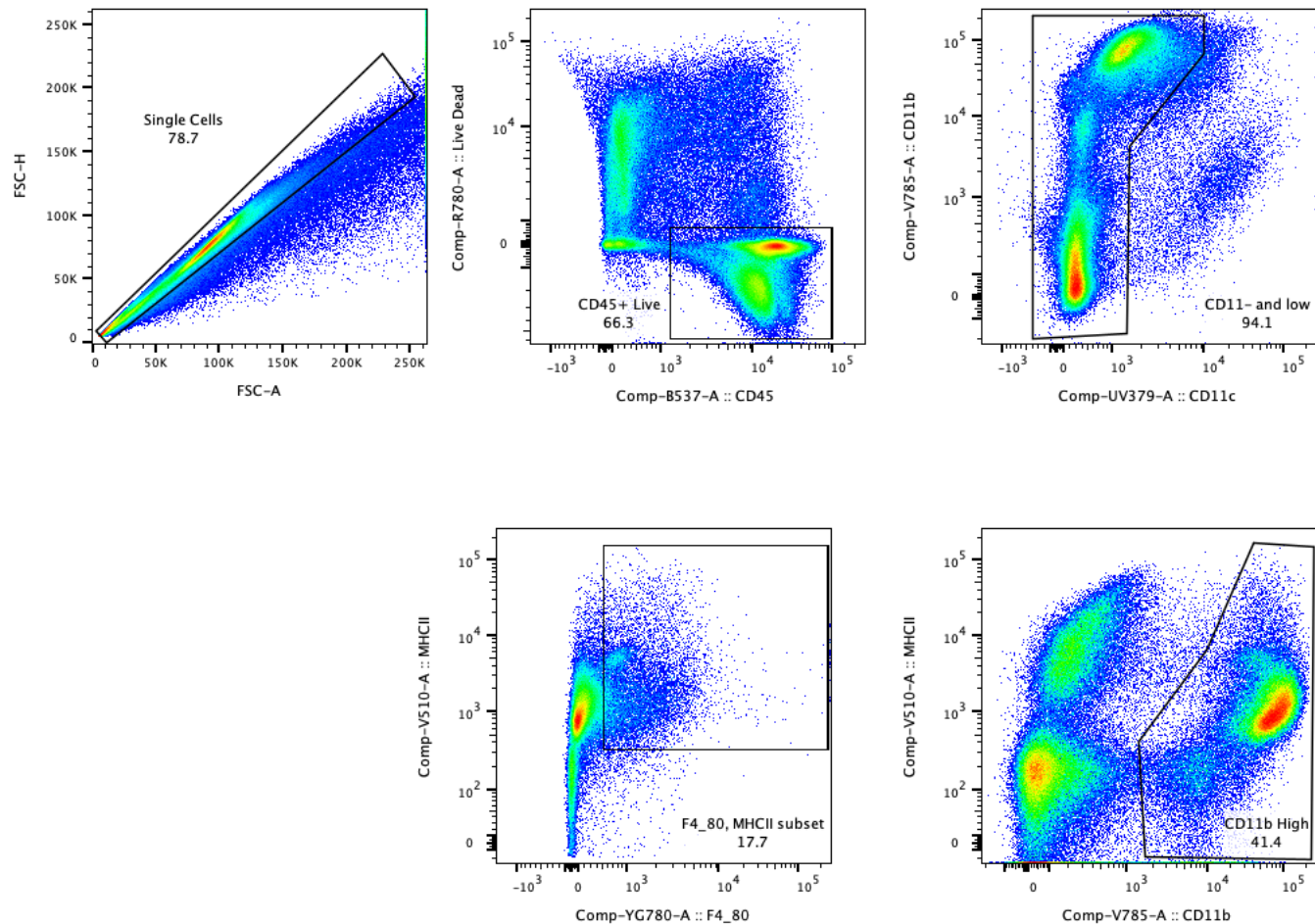

**Figure S1: Flow cytometry gating strategy.** Gating strategy used to filter macrophages.

Supplement: S1 Fig — Gating strategy used to filter macrophages. (PDF) [file pntd.0012585.s001.pdf]
